# Composition-Controlled Photocatalytic and Antibacterial Performance of ZnO-ZnS Nanocomposite Catalysts Synthesized by Solid-State Ion Exchange

**DOI:** 10.3390/molecules31061010

**Published:** 2026-03-17

**Authors:** Joanna Wojtas, Viktor Zinchenko, Renata Wojnarowska-Nowak, Dana Popescu, Anna Żaczek, Igor Magunov, Pavel Doga, Anton Babenko, Sergii Pavlov, Yaroslav Bobitski, Joanna Kisała

**Affiliations:** 1 Faculty of Medicine, Collegium Medicum, University of Rzeszow, Kopisto 2a Ave., 35-315 Rzeszow, Poland; jowojtas@ur.edu.pl (J.W.); azaczek@ur.edu.pl (A.Ż.); 2 A.V. Bogatsky Physico-Chemical Institute of National Academy of Sciences of Ukraine, 65080 Odesa, Ukraine; vfzinchenko@ukr.net (V.Z.); igmagua@ukr.net (I.M.); dogapavel@gmail.com (P.D.); 3 Faculty of Exact and Technical Sciences, University of Rzeszow, Pigonia 1 Str., 35-310 Rzeszow, Poland; rwojnarowska@ur.edu.pl (R.W.-N.); ybobytskyy@ur.edu.pl (Y.B.); 4 National Institute of Materials Physics, Atomistilor 405A, 077125 Magurele, Romania; dana.popescu@infim.ro; 5 Faculty of Chemistry and Pharmacy, Odesa I.I. Mechnikov National University, 65082 Odesa, Ukraine; anton.octane.sr@gmail.com; 6 Department of Biomedical Engineering and Optic-Electronic Systems, Vinnytsia National Technical University, 21021 Vinnytsia, Ukraine; psv@vntu.edu.ua

**Keywords:** nanostructured materials, ZnO–ZnS nanocomposites, photocatalysis, optical properties, antibacterial activity

## Abstract

Zinc oxide (ZnO) and zinc sulfide (ZnS) nanocomposites represent promising multifunctional photocatalysts due to their complementary band structures and synergistic charge separation. ZnO–ZnS nanocomposites with varied ZnS content were synthesized to elucidate the composition–structure–property relationships governing their multifunctional performance. Structural characterization using XRD, SEM/EDS, Raman spectroscopy, and XPS confirmed the coexistence of wurtzite crystalline phases of ZnO and ZnS. SEM analysis revealed ZnS fine deposition on the ZnO surface. XPS measurements showed a gradual increase in the amount of ZnS on the ZnO surface with increasing sulfide content and a shift in the valence band maximum from 2.32 eV (pure ZnO) to 0.77 eV (pure ZnS). Optical measurements (IR, UV–Vis diffuse reflectance, photoluminescence) demonstrated that, despite the evolution of vibrational and luminescence features characteristic of ZnS, the apparent band gap remained nearly constant at 3.16–3.18 eV across the series. Photocatalytic methylene blue (MB) degradation followed pseudo-first-order kinetics, peaking for ZN_2 (1% ZnS, k_app_ = 103 × 10^−3^ min^−1^), which is 1.7 times higher than for pure ZnO. This enhanced performance is consistent with an S-scheme-like heterojunction that facilitates electron migration to the ZnS conduction band while retaining ZnO valence band holes for oxidation. Scavenging experiments confirmed that electrons dominate MB degradation (k_app_ up to 185.1 × 10^−3^ min^−1^ with EDTA/t-BuOH/Ar), outperforming hole-mediated pathways. Antibacterial assays against *Staphylococcus aureus* revealed good antimicrobial activity for all nanoparticles. The nanocomposite’s antibacterial activity was similar across all samples and was only slightly lower than that of pure ZnS and ZnO.

## 1. Introduction

Zinc oxide (ZnO) and zinc sulfide (ZnS) are robust II–VI semiconductor materials with wide band gaps, favourable stability, and relatively low toxicity, making them attractive for applications in photocatalysis, antimicrobial coatings, sensors, and biomedical devices. Individually, ZnO exhibits strong photocatalytic activity under UV/near-UV light, but suffers from rapid recombination of charge carriers and limited visible-light response; ZnS, though less utilized, has advantages in forming heterojunctions and enhancing the separation of photogenerated charges when paired with ZnO. Combining ZnO–ZnS into heterostructured or composite systems has emerged as a promising strategy to overcome these limitations through band alignment, improved light absorption, and enhanced surface reactivity.

Several recent studies illustrate this trend. Shreya et al. demonstrated that ZnO@ZnS core–shell heterostructures significantly improved degradation efficiency of malachite green and rhodamine B dyes over their single-component analogues [1]. Sunaina et al. synthesized ZnO–ZnS heterostructures via a solvent-free route by thermal annealing of a solid-state mixture of ZnO and thiourea (a sulphur source) which results in a ZnO–ZnS core–shell [2]. The formation of heterostructure results in a band-gap reduction in comparison to the bare ZnO and ZnS nanoparticles. Further, these ZnO–ZnS heterostructures were utilized as a photocatalyst for the degradation of *p*-nitrophenol and organic dye (methyl orange) under light exposure (>390 nm). The obtained heterostructures show higher photodegradation efficiency than that of pure ZnO and ZnS. Salaheldin et al. (2024) produced biosynthesized ZnO–ZnS nanocomposites which removed ~87.5% of methylene blue dye and showed dose-dependent cytotoxicity against cancer cells [3]. Meanwhile, a composite of ZnO–ZnS with activated carbon derived from banana peel showed strong antibacterial activity against *Escherichia coli* and *Staphylococcus aureus* in addition to pollutant degradation performance [4]. These works highlight how both composition and morphology critically influence the functional properties of ZnO–ZnS systems under realistic conditions.

Beyond photocatalysis, ZnO nanostructures have been intensively explored as antimicrobial coatings for medical devices. The review by Puspasari et al. outlines advanced ZnO-based coatings and their mechanisms of bacterial inactivation, emphasizing the importance of nanoscale surface features and oxide chemistry in achieving biocompatibility without unwanted cytotoxicity [5].

*Staphylococcus aureus*, a Gram-positive bacterium, infects diverse human cells, organs, and tissues, causing severe infections with high mortality rates—for instance, it accounted for 18.9% of surgical-site infections in hospital settings [6]. This pathogen exhibits remarkable resistance mechanisms that complicate treatment. The rise of resistant strains, particularly methicillin-resistant *S. aureus* (MRSA), severely hampers eradication efforts in healthcare facilities [7,8,9]. *S. aureus* produces enzymes like proteases, lipases, and nucleases that facilitate extraction of nutrients from host tissues, fueling bacterial proliferation [10]. It evades host immunity through cytotoxins—especially hemolysins—and other virulence factors [11]. Additionally, biofilm formation provides a key survival strategy; these matrix-encased communities resist mechanical shear, phagocytosis, and antibiotic penetration due to poor drug diffusion [12,13]. Nanoparticles—metallic or semiconducting—offer a promising alternative for combating antibiotic-resistant *S. aureus* strains.

In this work, we synthesized ZnO–ZnS composites with varying ZnS content and investigated their structural, optical, photocatalytic, and antibacterial properties. Our approach employs comprehensive characterization methods (XRD, SEM/EDS, Raman, XPS, IR, UV-Vis diffuse reflectance, and photoluminescence) to relate microstructure and phase composition to photocatalytic performance. Photocatalytic activity is assessed through the degradation of methylene blue (MB). MB serves as a widely adopted model pollutant in photocatalytic degradation studies due to its well-characterized cationic structure, intense visible absorbance at 664 nm, and stable properties that facilitate reliable kinetic monitoring [14,15]. The antibacterial efficacy is tested against *Staphylococcus aureus*. This detailed correlation between composition, structure, photocatalytic, and antibacterial performance provides new insights into optimizing the composition of ZnO–ZnS nanocomposites for multifunctional applications.

## 2. Results and Discussion

### 2.1. Structural and Optical Characterization

#### 2.1.1. XRD Analysis and Phase Identification

**Figure 1:**
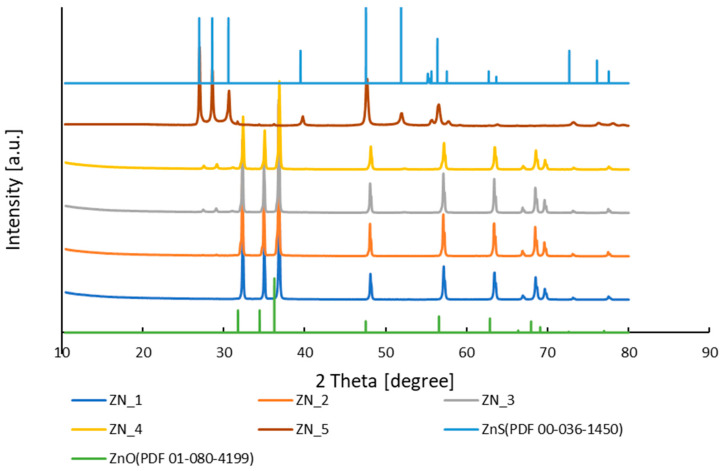
Diffraction spectra of samples of ZnO–ZnS systems (indicated by the corresponding colour: ZN_1—blue; ZN_2—orange; ZN_3—grey; ZN_4—yellow) and reference pattern for ZnS (light blue, PDF 00-036-1450) and ZnO (green, PDF 01-080-4199).

The XRD patterns revealed high crystallinity across all samples, characterized by sharp and intense diffraction peaks (Figure 1). For pure ZnO, characteristic peaks appeared at 2θ values of 31.77°, 34.42°, 36.25°, 47.54°, 56.60°, 62.86°, 66.48°, 67.91°, 69.10°, 72.85°, and 76.99°, corresponding to the (100), (002), (101), (102), (110), (103), (200), (112), (201), (004), and (202) planes of the hexagonal wurtzite structure (JCPDS No. 01-080-4199, space group P63mc), respectively. In samples ZN_1–ZN_4, diffraction peaks matched those of wurtzite ZnO. ZnS wurtzite phase peaks were identified at 2θ = 27.67°, 29.09°, 31.28°, 48.17°, 52.55°, 57.23°, 63.46°, 73.34°, and 77.67°, assigned to the (100), (002), (101), (110), (103), (112), (202), (203), and (210) planes (JCPDS No. 00-036-1450, space group P63mc), using the ICDD PDF-2 database [16] and Crystallographic Open Database [17] for phase identification. Sample ZN_1 exhibited a pure ZnO phase; ZN_2 showed a minor ZnS peak at 2θ ≈ 29.7°, while ZN_3 and ZN_4 displayed distinct ZnS peaks at 2θ = 27.67°, 29.62°, and 40.72° alongside ZnO, confirming ZnO-ZnS nanocomposite formation; ZN_5 consisted exclusively of wurtzite ZnS.

#### 2.1.2. Morphology and Composition by SEM/EDS

EDS analysis (Table 1, Figure S1) confirms the presence of Zn, O and S in the ZnO–ZnS samples. However, the S-peak in the ZN_1 and ZN_2 spectra is on the threshold level. At the same time, with an increase in the ZnS content specified by the synthesis, the S content increases, which is entirely logical. Since the EDS analysis is carried out at a certain depth of the sample (up to one micron), it shows a smaller proportion of atoms on the surface, compared to deeper layers.

**Figure 2:**
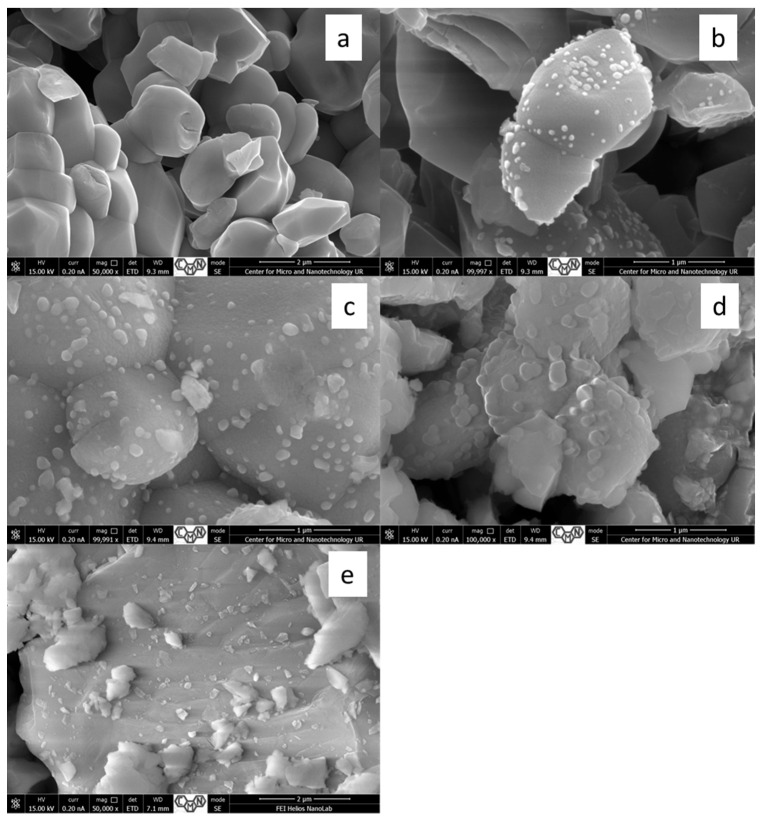
Electron SEM micrographs of samples of the ZnO–ZnS system: ZN_1 (**a**), ZN_2 (**b**), ZN_3 (**c**), ZN_4 (**d**), ZN_5 (**e**).

SEM micrographs show (Figure 2) a gradual increase in the size and number of ZnS nanoparticles covering the surface of large ZnO particles. At the same time, the shape of the nanoparticles gradually changes towards a more flattened shape due to their coalescence. As can be seen in Figure 2, in the case of composites (ZN_2 to ZN_4), ZnO particles are covered with fine ZnS particles, while on the surface of the ZN_1 sample they are not observed.

#### 2.1.3. Raman Spectroscopy Phase Confirmation

**Figure 3:**
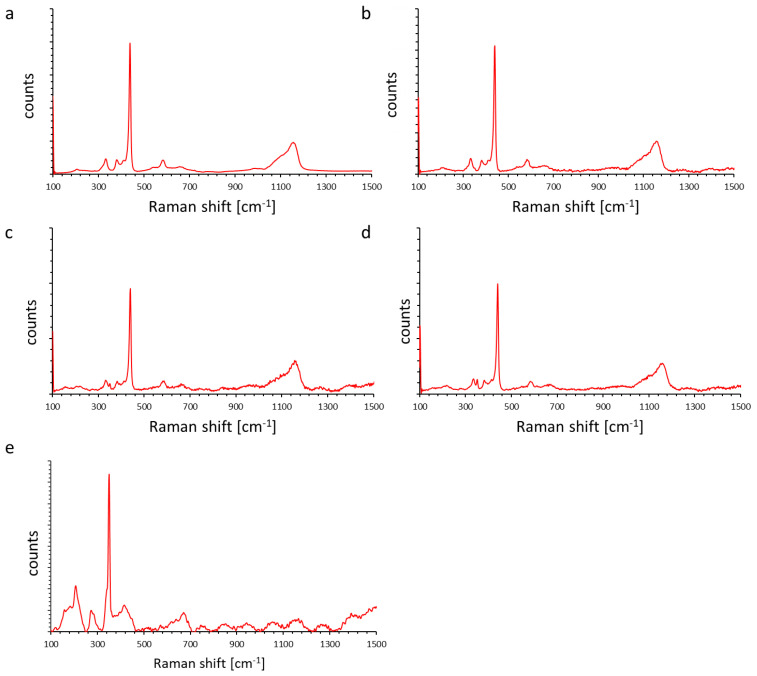
Raman spectra of ZN_1 (**a**), ZN_4 (**b**), ZN_3 (**c**), ZN_4 (**d**), and ZN_5 (**e**) samples (λ = 488 nm).

Raman spectroscopy was used to assess the structural quality and phase composition of the ZnO, ZnS, and ZnO–ZnS composites (Figure 3). All samples exhibited the characteristic vibrational modes of wurtzite ZnO, with the dominant E_2_(high) phonon mode at ~439 cm^−1^, indicating high crystallinity and the absence of parasitic phases [18,19,20]. The values of full width at half maximum (FWHM) of E_2_(high) mode are 7–8 cm^−1^, which indicates a good wurtzite ZnO structure. Other ZnO features included ~100 cm^−1^ (E_2_(low)), 381 cm^−1^ (A_1_(TO)), ~409–413 cm^−1^ (E_1_(TO)), and ~584–587 cm^−1^ (A_1_(LO)/E_1_(LO)). The multi-phonon scattering modes are presented at ~332 cm^−1^ (E_2_(high)–E_2_(low)) and ~656–664 cm^−1^ (2(E_2_(high)–E_2_(low)).

Upon increasing ZnS content, additional bands emerged at ~274–280 cm^−1^ and ~349–351 cm^−1^ corresponding to ZnS transverse optical (TO) and longitudinal optical (LO) first-order phonon modes [21,22,23]. The line at ~392–395 cm^−1^ belongs to the low-frequency region, caused by acoustic overtones and second-order transverse optical (TO + LA) modes [22,24]. The second-order LO mode located at ~658 cm^−1^ is overlapped with 2(E_2_(high)–E_2_(low)) mode of ZnO.

The relative intensity of ZnS bands scaled with ZnS loading (1 → 15 wt.%), while the intensity of ZnO’s E_2_ (high) decreased (from ~20,000 a.u. in pure ZnO to ~9500 a.u. in the 90% ZnO composite), reflecting surface coverage by Raman-weaker ZnS and reduced sampling volume of ZnO. Raman mapping confirmed high homogeneity for low ZnS contents (1–5 wt.%), whereas mild heterogeneity appeared at higher ZnS fractions due to local ZnS enrichment. Detailed identification of individual bands is presented in Table 2. These assignments are consistent with reported values for ZnO (E_2_(high) ~437–440 cm^−1^; A_1_/E_1_(LO) ~575–590 cm^−1^) and ZnS (LO ~350 cm^−1^; TO near 390–395 cm^−1^) in ZnO–ZnS nanocomposites.

Taken together, the Raman results confirm that the ZnO lattice remains intact after ZnS incorporation, with increasing ZnS modes evidencing composite formation. The absence of extra peaks supports the high purity and crystallinity of the ZnO–ZnS systems.

Measurements in mapping mode were performed for each sample (102 point—sample ZN_1; 138—sample ZN_2; 159—sample ZN_3; 116—sample ZN_4; 93—sample ZN_5) (Figure S4). Samples ZN_1, ZN_2, and ZN_5 have good material homogeneity, which is demonstrated by consistent spectra for all measurement points. Samples ZN_3 and ZN_4 are more inhomogeneous. In different measurement locations, slightly different intensities of the bands assigned to ZnS were recorded.

#### 2.1.4. IR Transmittance Spectroscopy

**Figure 4:**
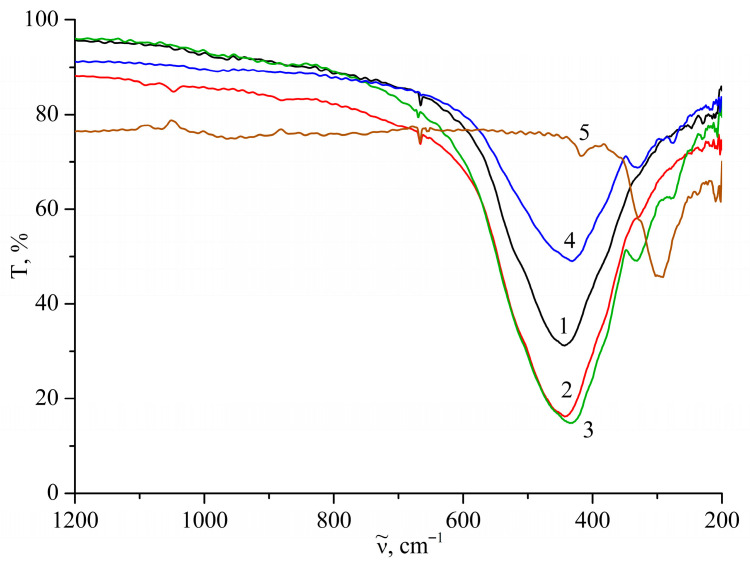
IR transmittance spectra of ZnO–ZnS system samples: 1—ZN_1 (black); 2—ZN_2 (red); 3—ZN_3 (green); 4—ZN_4 (blue); 5—ZN_5 (brown).

The IR spectra of the ZnO–ZnS systems change significantly with increasing ZnS content. Thus, the main band corresponding to the lattice vibrations (Zn–O bonds) of the substrate shifts noticeably towards lower values of $\tilde{\nu }$; at the same time, the absorption bands of the ZnS impurity appear and intensify, but at slightly higher values of $\tilde{\nu }$ than those of the individual ZnS compound (Table 3). The intensity of the absorption band (Figure 4) of Zn–O lattice vibrations changes non-monotonically, first increasing and then decreasing with increasing additive content. This change is characteristic of other spectral parameters of the studied system and is evidence of a deep interaction between its components.

Consequently, the deepest absorption in the far-infrared spectral range falls on the sample of the system containing 90 wt.% ZnO and, accordingly, 10 wt.% ZnS. Perhaps this composition corresponds to the largest deviation from ideal behaviour in the system, or its maximum disorder.

#### 2.1.5. Optical Properties

**Figure 5:**
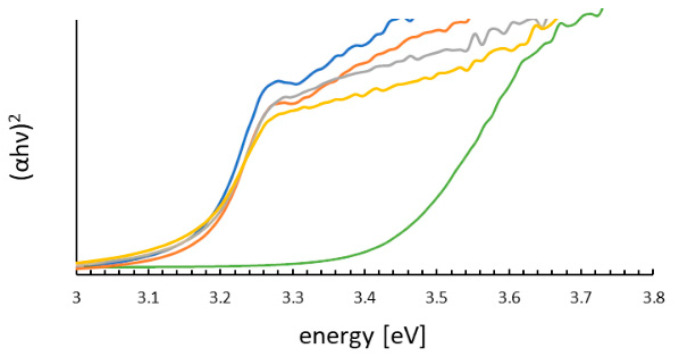
Tauc plots of the ZnO–ZnS system samples (ZN_1—blue; ZN_2—orange; ZN_3—grey; ZN_4—yellow; ZN_5—green).

The optical properties of the prepared samples were measured by diffuse reflectance UV-Vis spectra (DRS) (Figure S2). The Tauc plot (Figure 5) calculated from DRS shows that the band gaps for all materials are similar and in the range of 3.16–3.18 eV. ZnO is a semiconductor with a band gap of approximately 3.2 eV, and ZnS has wide band gap of 3.41 eV. The strain at the nanocomposite boundary slightly reduces the natural band gap. One-band-edge absorption is observed in all catalysts at ca. 390 nm. The band-gap energies are summarized in Table 4.

Sundararajan et al. [25] investigated the optical properties of ZnO–ZnS nanocomposites with ZnO:ZnS molar ratios of 25:75, 50:50, and 75:25, reporting band-gap energies that increased from 3.07 eV to 3.11 eV with higher ZnO content. In contrast, the present ZnO:ZnS nanocomposites maintained nearly constant molar ratios close to 1:0 (ZN_1), 1.22:0.01 (ZN_2), 1.11:0.1 (ZN_3), and 1.04:0.15 (ZN_4), resulting in negligible differences in band-gap energies (3.16–3.18 eV) across the series.

In these ZN_2-ZN_4 composites, the nearly constant apparent band gap reflects that both end members (ZN_1 and ZN_5) are wide-band-gap wurtzite semiconductors (≈3.2 eV and 3.4 eV, respectively) and that the heterostructure is realized mainly as a surface ZnS on a ZnO core, with limited bulk alloying. XRD and Raman show that the two phases remain structurally distinct (no new mixed phase, only ZnO and ZnS peaks), indicating that the composites are physical heterostructures rather than bulk solid solutions; as a result, the optical edge is dominated by the ZnO-like transition around 3.16–3.18 eV over most of the series. SEM images show that ZnS forms fine crystals on the ZnO particles. Although interfacial interactions between ZnO and ZnS may induce local electronic perturbations, these effects are likely too small to produce a distinct shift in the Tauc-derived optical band gap.

#### 2.1.6. X-Ray Photoelectron Spectroscopy

X-ray photoelectron spectroscopy (XPS) is used to investigate the surface composition and chemical state. All binding energy (B.E.) values in XPS spectra were calibrated using the C1 s line at 284.6 eV. Survey spectra of the ZN_1–ZN_5 (Figure S4) powders show Zn, O, C and S as the only detectable elements, in agreement with the nominal compositions of ZnO, ZnS and ZnO–ZnS composites. The S 2p intensity increases systematically from ZN_1 to ZN_5, whereas the Zn/S intensity ratio decreases (I(Zn)/I(S) ≈ 13; 6.75; 1; 0.94 and 0.36 for ZN_1–ZN_5, respectively) (Table S2), confirming the progressive enrichment of ZnS in the series.

High-resolution Zn 2p spectra of ZN_1–ZN_4 can be deconvoluted into two components, while ZN_5 shows a single contribution. For ZN_1–ZN_4 the main component is located at BE ≈ 1020.2–1020.7 eV and the minor one is at ≈ 1021.4 eV; ZN_5 exhibits only the low-BE component at 1020.7 eV. All peaks are characteristic of Zn^2+^ species; no signature of metallic Zn is observed.

The Auger parameter (α) is widely used to distinguish between Zn oxidation states. It combines the Zn 2p_3/2_ binding energy and Zn LMM kinetic energy (Figure S5), minimizing effects of surface charging or work function shifts. For Zn-based systems, reference values are well established. The Auger parameter (α) is defined as (Equation (1))

(1) $$\alpha =BE\;\left(Zn\;2p_{3/2}\right)+KE\;(Zn\;LMM)$$

Using this KE and the fitted 2p_3/2_ binding energies, the Auger parameters were calculated for both Zn 2p components (Table S1). Because the same C 1s correction is applied to all regions, any rigid charging shift cancels in the sum BE + KE, so α is independent of the adventitious-carbon calibration. Across the ZN_1–ZN_4 series, the low-BE component (≈1020.2–1020.7 eV) yields α ≈ 2008.8–2009.2 eV, while the high-BE component (≈1021.4 eV) gives α ≈ 2010.0–2010.4 eV. ZN_5, which consists nominally of pure ZnS, exhibits only the low-BE component with α ≈ 2009.0 eV.

Comparison with reference α values for Zn compounds indicates that α ≈ 2009.0–2009.2 eV is typical of Zn(OH)_2_ and hydroxylated/carbonate-like ZnO surface species [26], whereas α ≈ 2010.0–2010.4 eV is consistent with lattice Zn^2+^ in ZnO-type environments [27,28], and overlaps with the lower part of the ZnS range reported in the literature [29,30] for ZnO–ZnS nanocomposites. Therefore, we assign the low-BE Zn 2p component (component 1) to Zn^2+^ in surface-hydroxylated ZnO/Zn(OH)_2_-like environments and the high-BE component (component 2) to lattice Zn^2+^ in the ZnO/ZnS framework. The fraction of lattice Zn^2+^ (component 2) decreases from ZN_1 to ZN_5, consistent with the increasing contribution of ZnS and with the S 2p behaviour described below. Although ZN_5 is nominally pure ZnS, surface oxidation leads to hydroxylated ZnO-like species dominating the Zn 2p and Auger parameter signatures.

**Figure 6:**
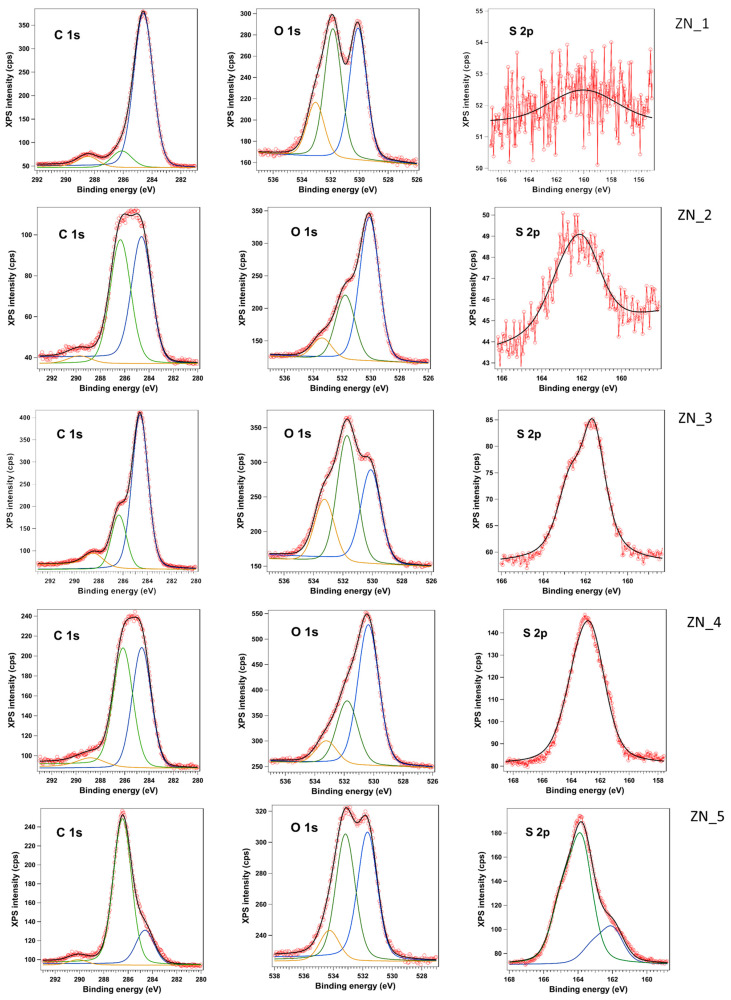
High-resolution X-ray photoelectron spectroscopy (XPS) measurements on samples ZN_1-ZN5. The experimental data are shown as red dots/lines, while the fit is represented by the black line. The C 1s and O 1s spectra could be deconvoluted into three components.

The S 2p region of ZN_2–ZN_5 is dominated by a doublet with S 2p_3/2_ at 161.6–162.6 eV, characteristic of S^2−^ in ZnS (Figure 6). No significant contributions are observed at higher binding energy (≈168–170 eV), indicating the absence of sulfate-type species. The systematic growth of the S 2p intensity and surface atomic percentage from ZN_1 to ZN_5 corroborates the incorporation of ZnS into the ZnO matrix and the formation of ZnO–ZnS nanocomposites. The S 2p signal in ZN_1 is extremely weak, and the apparent feature at 159.7 eV is below the reliable detection threshold and does not correspond to sulfide.

All samples exhibit a pronounced C 1s signal arising from adventitious carbon (Figure 6). Deconvolution of the C 1s peak reveals components at ≈284.6 eV (C–C/C–H), 286.1–286.5 eV (C–O/C–OH/C–O–C) and 288.3–290.1 eV (C=O/O–C=O), in proportions that vary moderately between samples.

The O 1s spectra can be fitted with up to three components: a main peak at ≈530.1 eV assigned to O^2−^ in ZnO (Figure 6), a second contribution at 531.7–531.9 eV related to hydroxyl or carbonyl oxygen (surface Zn–OH and C=O), and a high-BE shoulder at ≈533–534 eV due to adsorbed water or molecular oxygen.

XPS confirms that all samples contain Zn^2+^ exclusively, with no detectable metallic Zn (Figure S6). The Auger-parameter analysis shows that the surface is composed of a mixture of hydroxylated ZnO/Zn(OH)_2_-like species (α ≈ 2009 eV) and lattice Zn^2+^ in the ZnO–ZnS framework (α ≈ 2010–2010.4 eV). The clear S^2−^ signal at 161.6–162.6 eV and the evolution of the Zn/S intensity ratio demonstrate the progressive formation of ZnO–ZnS nanocomposites as the nominal ZnS content increases from ZN_1 to ZN_5.

The VBM values extracted from the valence band spectra exhibit an overall downward shift from ZN_1 to ZN_5, consistent with the increasing ZnS content in the series (Table 4). The VBM decreases from 2.32 eV in ZN_1 to 0.77 eV in ZN_5, reflecting the known deeper valence band of ZnS with respect to ZnO (Figure S7).

A slight deviation from the monotonic trend is observed for ZN_3, which displays a lower VBM (0.91 eV) than ZN_4 (1.14 eV). This behaviour correlates with the surface composition obtained from XPS measurements: ZN_3 exhibits the highest relative S 2p intensity and the lowest fraction of lattice Zn^2+^ among the mixed samples, indicating that its surface is more ZnS-rich than that of ZN_4. In contrast, ZN_4 shows a larger contribution from ZnO-like lattice Zn^2+^ species at the surface. Since the VBM position is highly surface-sensitive, the deeper VBM in ZN_3 reflects its more pronounced ZnS-like surface character. Overall, the VBM evolution supports the formation of ZnO–ZnS nanocomposites with surface-dependent band alignment, where ZnS-rich surfaces drive the valence band to higher binding energies while ZnO-rich surfaces shift it closer to the Fermi level.

The strong composition-dependent changes in valence band position from XPS (VBM shift from 2.32 eV for ZnO to 0.77 eV for ZnS) confirm that band alignment and interfacial band bending are significantly modified even though the optical gap extracted from DRS remains almost unchanged (Table 4). This result supports the idea that photocatalytic enhancement is driven by S-scheme-like charge separation rather than by band-gap narrowing.

#### 2.1.7. Photoluminescence Spectroscopy

**Figure 7:**
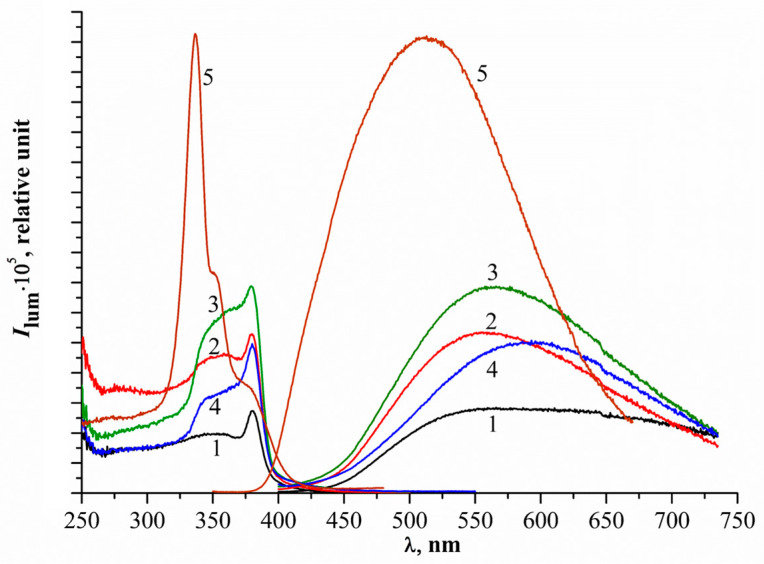
Photoluminescence spectra of catalysts (excitation spectra on the left side, emission spectra on the right side), excitation 380 nm, slits 1–5 nm: 1—ZN_1 (black); 2—ZN_2 (red); 3—ZN_3 (green); 4—ZN_4 (blue); 5—ZN_5 (brown).

PL spectra are used to determine the separation ability and recombination rate between photogenerated electrons and holes in semiconductor material structures. The excitation and emission spectra (Figure 7) differ significantly in the shape and position of the luminescence band maxima (Table 5). The strong peaks were observed at 461, 555, 564, and 593 nm. Weak peaks at 375 nm also appear after 300 nm excitation (Figure S8).

The peaks at 555 and 564 nm correspond to green emission (ZN_1, ZN_2, ZN_3), whereas 593 nm corresponds to orange emission (ZN_4). The sample ZN_5 reveals blue emission. The green emission can be attributed to defects, specifically singly ionized oxygen vacancies [31,32].

This implies that ZnO has the ability to inhibit the recombination of electrons and holes, thereby helping to enhance the photocatalytic effects of the material. Moreover, the emission peak at 564 nm of ZnO may be assigned to the oxygen vacancy defect states of ZnO. Meanwhile, the emission peak of ZnS located at 460 nm could be assigned to the radiative recombination involving defect or vacancy states at the surface of the ZnS nanocrystals [33].

The UV emission in the range of 379–420 nm is related to the recombination of photoelectrons in a conduction band with the holes in a valence band [34]. Visible emissions in ZnO arise from intrinsic crystal defects, such as oxygen vacancy (V_O_), zinc vacancy (V_Zn_), interstitial oxygen (O_i_), and interstitial zinc (Z_i_) [35]. It is generally believed that the surface oxygen vacancies, whose presence is shown as green emission (495–566 nm) and yellow emission (566–589 nm), could be assigned to the surface and interstitial oxygen vacancies, respectively [36]. All samples show a relatively high green emission, which is attributed to recombination of one or two electrons with the surface oxygen vacancy (V_Osurface_). The yellow emission is involved with recombination of delocalized electrons and holes in oxygen interstitials [37].

The increase in emission peaks in samples ZN_2, ZN_3, and ZN_4 suggests that the recombination rate of photogenerated charge carriers is higher than in ZN_1 and results in slower charge transfer and separation rates [38,39]. However, in heterogeneous ZnO–ZnS systems, the PL response reflects not only radiative interband recombination but also multiple defect-related transitions, particularly those associated with oxygen vacancies in ZnO and surface defect states in ZnS. Therefore, PL intensity should not be interpreted solely as a direct measure of the overall recombination rate. In our samples, the visible emission bands at 555–593 nm and 461 nm attributed to defect states (surface oxygen vacancies in ZnO and vacancy-related centres in ZnS) may also promote surface adsorption and interfacial charge transfer. Consequently, the increased PL intensity observed for some composites (ZN_2–ZN_4) likely reflects a higher density of radiative defect centres rather than simply increased detrimental bulk recombination. This interpretation is consistent with the photocatalytic results (described below), as ZN_2 shows the highest MB degradation rate despite stronger defect-related PL than ZN_1, suggesting that suitably distributed defect states, together with the S-scheme-like ZnO–ZnS junction, may facilitate the generation of reactive species. At the same time, a minor contribution from interfacial strain cannot be excluded, as such strain may slightly perturb the local electronic structure. Similarly, defect states associated with the heterointerface or surface vacancies may affect the absorption tail and PL behaviour more strongly than the fundamental band-edge transition. Accordingly, the nearly unchanged band-gap values most likely reflect the dominance of ZnO-related optical absorption, along with the limited sensitivity of diffuse-reflectance spectroscopy to subtle interface- and defect-induced changes in heterogeneous nanocomposites. By contrast, the strong composition-dependent shift in the valence-band maximum determined by XPS, from 2.32 eV for ZnO to 0.77 eV for ZnS, indicates substantial modification of band alignment and interfacial band bending even though the optical gap derived from DRS remains nearly constant. These findings support the view that the enhanced photocatalytic performance is governed mainly by S-scheme-like charge separation rather than by band-gap narrowing. Therefore, the PL results should be treated as complementary to the kinetic data, highlighting changes in defect chemistry and interfacial structure rather than serving as a stand-alone quantitative indicator of charge-carrier recombination.

### 2.2. Photocatalytic Activity Evaluation

**Figure 8:**
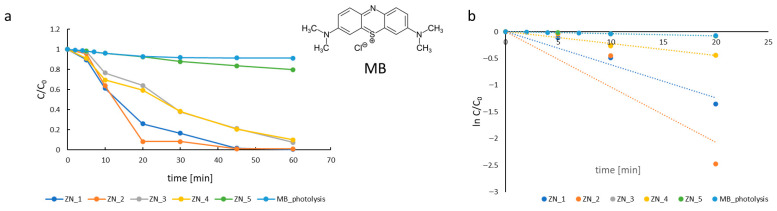
Kinetic curves of methylene blue degradation rate in the presence of ZnO–ZnS system samples (ZN_1—blue; ZN_2—orange; ZN_3—grey; ZN_4—yellow); insert—MB structure (**a**). Kinetic curves of methylene blue degradation rate in the presence of ZnO–ZnS system samples in semi-logarithmic coordinates (ZN_1—blue; ZN_2—orange; ZN_3—grey; ZN_4—yellow) (**b**).

The photocatalytic performance of the materials (ZN_1–ZN_5) was evaluated via the degradation of an aqueous methylene blue (MB) solution. To elucidate the roles of photogenerated charge carriers in the degradation process, a series of trapping experiments was conducted under different conditions: (a) in the presence of air, (b) in the presence of oxidative species and hole scavengers (tert-butanol, EDTA), and (c) in the presence of an electron scavenger (KClO_3_). Experiments (b) and (c) were carried out under an argon atmosphere to minimize the influence of dissolved oxygen. Experiment (a) was performed under weakly acidic conditions (pH 5), whereas experiments (b) and (c) were conducted at pH 6. The decay of MB concentration in photocatalytic process (a) is shown in Figure 8a. The apparent photodegradation rate constant (k_app_) was determined for each catalyst by assuming pseudo-first-order kinetics, according to Equation (2) (Figure 8b).

(2) $$ln\frac{C}{C_{0}}=-k\cdot t$$

where k_app_ is the apparent rate constant; C_0_ and C are the initial concentration and concentration at time t.

The degradation rate constants are summarized in Table 6. The ZN_2 sample exhibited the highest catalytic performance (k_app_ = 103.3 × 10^−3^ min^−1^). Pure ZnO (ZN_1) showed a lower rate, while ZN_3 and ZN_4 achieved identical rates of 22.2 × 10^−3^ min^−1^. These results demonstrate that low ZnS content (1 wt.%) enhances photocatalytic activity relative to pure ZnO, whereas higher ZnS loadings (10–15 wt.%) suppress performance. This trend correlates with the conduction and valence band potentials of ZN_3 and ZN_4 (Table 4), indicating suboptimal band alignment in ZnS-rich nanocomposites.

In this system the reactive species could be generated [40]:

$$Cat\;\overset{h\nu }{\to }\;Cat\left(e^{-},\;h^{+}\right)$$

$$O_{2}+e^{-}\to O_{2}^{•-}$$

$$H^{+}+e^{-}\to H^{•}$$

$$O_{2}^{•-}+H^{•}\to {HOO}^{-}$$

$${HOO}^{-}+H^{+}\to HOOH$$

$${HOO}^{-}+h^{+}\to {HOO}^{•}$$

$$HOOH+e^{-}\to {HO}^{•}+{HO}^{-}$$

$${MB}^{+}+h^{+}\to {MB}^{+•}$$

$$H_{2}O+h^{+}\to {HO}^{•}+H^{+}$$

The standard redox potentials for O_2_/O_2_^•−^ (−0.33 V) indicate that superoxide radicals can be generated by conduction band electrons [41]. The hydroxyl radical formation potential from water (E_(H2O/HO•)_ = +2.38 V) [42] aligns with ZnO’s valence band position, enabling –OH generation via hole oxidation of adsorbed water. Direct hole oxidation of MB is also feasible (E_MB2+/MB+_ = +1.25 V vs. NHE) [43].

The superoxide radical (O_2_^•−^) is the conjugate base of hydroperoxide (HOO^•^, pKa = 4.88) [44]. At pH 4–6, both species coexist, with protonation of O_2_^•−^ by H^+^ occurring diffusion-controlled (k = 5 × 10^10^ M^−1^ s^−1^). Thus, multiple ROS (O_2_^•−^, ^•^OH, H_2_O_2_, ^•^OOH) contribute to MB oxidative degradation.

**Figure 9:**
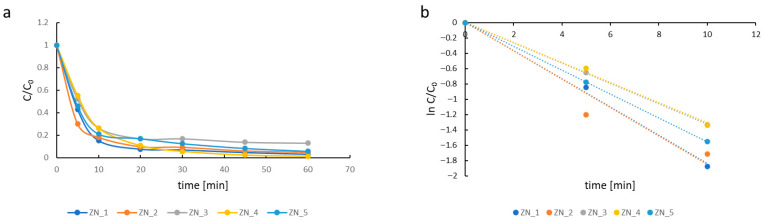
Kinetic curves of methylene blue degradation rate in the presence of EDTA as hole scavenger (ZN_1—blue; ZN_2—orange; ZN_3—grey; ZN_4—yellow) (**a**). Kinetic curves of methylene blue degradation rate in the presence of EDTA in semi-logarithmic coordinates (ZN_1—blue; ZN_2—orange; ZN_3—grey; ZN_4—yellow) (**b**).

To elucidate the dominant reactive species responsible for the observed photocatalytic activity, scavenging experiments were conducted using tert-butanol (t-BuOH) as a quencher for H^•^ and ^•^OH radicals, EDTA as a hole scavenger, and KClO_3_ as an electron scavenger. Figure 9a demonstrates that the combination of Ar, t-BuOH, and EDTA significantly suppressed MB degradation kinetics. Argon purging eliminated dissolved O_2_ from the reaction mixture, while t-BuOH effectively quenched both H^•^ and ^•^OH radicals. EDTA, acting as an efficient hole scavenger, prevented direct h^+^ oxidation of MB. Under these conditions, the enhanced catalytic performance observed across all samples must, therefore, be attributed to conduction band electrons possessing sufficient reducing potential for MB reduction E _(MB+/MB•)_ = −0.23 V [42] (noting that superoxide radical formation was precluded by O_2_ removal). Figure 9b confirms the high degradation efficiency maintained by all catalysts, with apparent rate constants ranging from 130 × 10^−3^ to 180 × 10^−3^ min^−1^ (Table 7). The following reactions govern this electron-mediated process [45,46]:

$$Cat\;\overset{h\nu }{\to }\;Cat\left(e^{-},\;h^{+}\right)$$

$$EDTA-2Na+h^{+}\to {\left[EDTA-2Na\right]}^{+•}$$

$$H^{+}+e^{-}\to H^{•}$$

$$H_{2}O+H^{•}\to {HO}^{•}+H_{2}$$

$$t-BuOH+h^{+}\to {\left[t-BuOH\right]}^{•}+H^{+}$$

$$t-BuOH+H^{•}\to {\left[t-BuOH\right]}^{•}+H_{2}$$

$$t-BuOH+{HO}^{•}\to {\left[t-BuOH\right]}^{•}+H_{2}O$$

$${MB}^{+}+e^{-}\to {MB}^{•}$$

To investigate the oxidative degradation pathway of MB, KClO_3_ was employed as an electron scavenger, with reactions conducted under an argon atmosphere to eliminate dissolved oxygen. Under these conditions, MB degradation could proceed solely via direct hole oxidation or through hydroxyl radicals generated by water oxidation at valence band holes.

**Figure 10:**
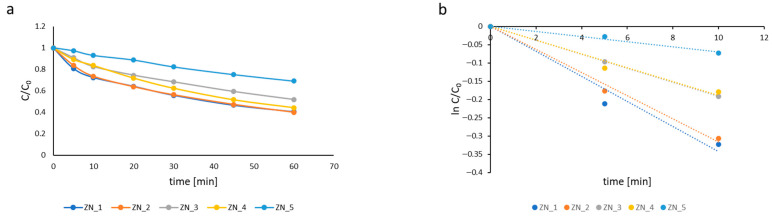
Kinetic curves of methylene blue degradation rate in the presence of KClO_3_ as electron scavenger (ZN_1—blue; ZN_2—orange; ZN_3—grey; ZN_4—yellow) (**a**). Kinetic curves of methylene blue degradation rate in the presence of KClO3 in semi-logarithmic coordinates (ZN_1—blue; ZN_2—orange; ZN_3—grey; ZN_4—yellow) (**b**).

**Figure 11:**
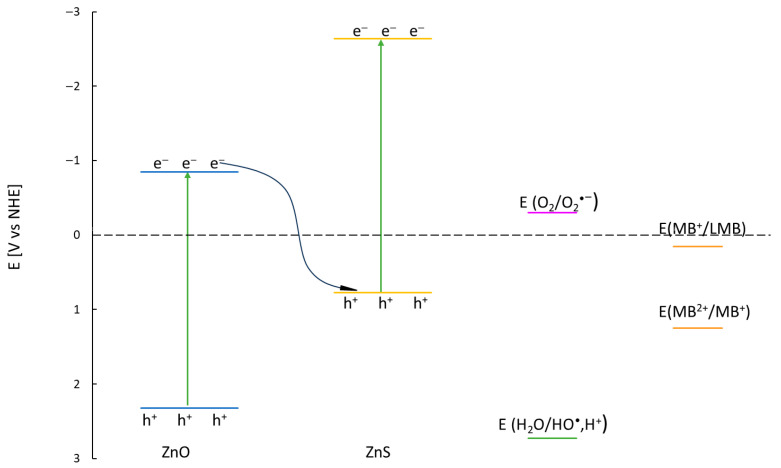
Schematic diagram of valence band and conduction band potential of ZnO and ZnS in relation to $E_{H_{2}O/HO^{\cdot },H^{+}}$ and $E_{O_{2}/O_{2}^{\cdot -}}$.

The apparent rate constants with the electron scavenger varied significantly across catalysts (Table 8). Increasing ZnS content in the ZN_2–ZN_4 nanocomposites progressively decreased degradation efficiency (Figure 10). A slight deviation from this monotonic trend between ZN_3 and ZN_4 correlates with the higher surface S content of ZN_3 (XPS data). ZN_5 exhibited the lowest k_app_, attributable to its insufficiently positive valence band potential for either hydroxyl radical generation or MB oxidation (Figure 11).

Reactions in the electron-scavenged system:

$$Cat\;\overset{h\nu }{\to }\;Cat\left(e^{-},\;h^{+}\right)$$

$${ClO}_{3}^{-}+e^{-}+6H^{+}\to {Cl}^{-}+3H_{2}O$$

$${MB}^{+}+h^{+}\to {MB}^{+•}$$

$$H_{2}O+h^{+}\to {HO}^{•}+H^{+}$$

In scavenging experiments, the observed decay of MB concentration occurs mainly as a result of the reaction with e^–^ and h^+^ trapped on the surface of the catalyst. ZnO valence band potential is +2.32 V vs. NHE, whereas ZnS E_VB_ = +0.77 V vs. NHE. When ZnO-ZnS nanocomposites are irradiated, the electron–hole pairs are generated on their conduction band (CB) and valence band (VB). Due to CB and VB edge potentials, the photogenerated electrons at ZnO CB migrate to ZnS VB, improving the spatial separation of charge carriers (Figure 9). Coulombic attraction of electrons in ZnO and holes in ZnS favours this charge transfer. Ultimately, the strong photogenerated electrons and holes are reserved in the CB ZnS and VB ZnO, respectively, whereas the weak photogenerated charge carriers undergo recombination. This type of heterojunction is consistent with S-scheme-like behaviour, possessing strong redox ability.

Photogenerated electrons migrate from ZnO’s conduction band (CB) to ZnS’s CB, while holes accumulate in ZnO’s valence band (VB). This charge separation increases electron density in ZnS’s CB and hole density in ZnO’s VB, enabling efficient generation of superoxide radicals from O_2_ (E_(O2/O2•−)_ = −0.33 V vs. NHE) [41] and direct hole oxidation of MB (E_(MB_^2+^_/MB_^+^_)_ = +1.25 V vs. NHE) [43]. ZnO’s sufficiently positive VB potential also permits water oxidation to hydroxyl radicals (^•^OH). Consequently, MB undergoes either direct hole oxidation or reaction with ^•^OH. However, scavenging experiments demonstrate that oxidative processes are significantly less efficient than electron-mediated reduction, indicating that conduction band electrons dominate MB degradation in this system, consistent with S-scheme-like behaviour.

### 2.3. Antibacterial Performance Assessment

**Figure 12:**
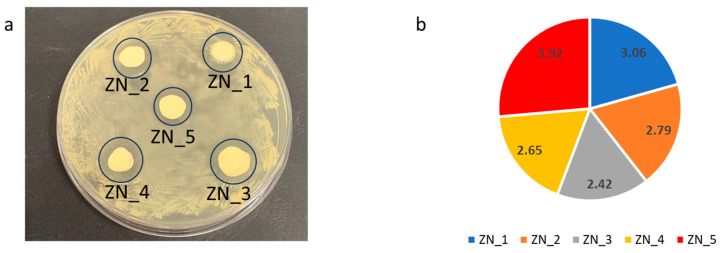
Visible inhibition of zones of growth around the applied tested nanoparticles (**a**). Comparison of the bactericidal activity of the tested nanoparticles (**b**), where the values represent the size of the inhibition zones in millimetres [mm].

The antibacterial activity of the nanoparticles (ZN_1 to ZN_5) was evaluated against the Gram-positive bacterium *Staphylococcus aureus* (ATCC 25923) using an agar diffusion assay. Distinct inhibition zones were observed for all samples (Figure 12a), with measured diameters ranging from 2.42 ± 1.40 mm to 3.92 ± 0.79 mm (Figure 12b). ZN_5 (pure ZnS) exhibited the strongest bactericidal effect, producing the largest inhibition zone (3.92 ± 0.79 mm). The composites ZN_2, ZN_3, and ZN_4 show slight decreases in activity (2.79 ± 1.05 mm, 2.42 ± 1.40, and 2.65 ± 0.86 mm, respectively).

These results indicate that all samples exhibit comparable antibacterial activity. This likely stems from well-established mechanisms, including reactive oxygen species (ROS) generation and nanoparticle deposition on bacterial surfaces or accumulation in the cytoplasm/periplasmic space, which disrupts cellular functions and compromises membrane integrity [47]. The proposed mechanism involves nanoparticles releasing Zn^2+^ ions that internalize into bacterial cells, disrupting enzymatic systems. These materials also generate ROS (O_2_^•−^, HO_2_^•−^, and ^•^OH), which inhibit biosynthesis of essential cellular components such as DNA, proteins, and lipids. Direct nanoparticle internalization and surface contact ultimately cause loss of cellular integrity. We propose that the synergistic effects of ROS production (as the Petri dishes were illuminated with a blue lamp) and nanoparticle accumulation—both on *S. aureus* surfaces and within the cytoplasm—account for the observed growth inhibition and bactericidal action.

Mediavilla et al. [4] demonstrated that ZnO–ZnS composites showed good antibacterial efficiency against *E. coli* and *S. aureus*. Comparable studies were conducted by Salaheldin et al. [3]. They evaluated the effectiveness of the ZnO–ZnS nanostructures in terms of their antibacterial activity against several pathogenic bacterial isolates. The results indicate that the ZnO–ZnS nanocomposite displayed significant antibacterial effects against *B. subtilis*, *E. coli*, *S. typhi* and *MRSA* bacteria (minimum inhibitory concentration (MIC) values of 50,000, 50,000, 5000 and 500 µg/mL, respectively).

At the same time, our ZnO–ZnS composites do not exhibit a pronounced compositional synergy in antibacterial performance, as all samples showed comparable inhibition zones against *S. aureus*, with only modest variations in activity. In contrast, Zn–Cu oxide systems have been reported [48] to display genuine synergistic enhancement of ROS-mediated antibacterial action at low Cu loading, where heterojunction formation and the combined effects of ROS generation and Cu^2+^ release lead to higher bactericidal efficiency than that of the individual oxides. This comparison helps place the present results in context and indicates that, in our system, the antibacterial response is better described as generally retained across compositions rather than strongly synergistically enhanced. In summary, our results show that ZnO-rich compounds retain the strongest antibacterial activity against *S. aureus*, whereas increased ZnS content compromises functionality, likely by reducing the exposure of active ZnO surfaces. These results highlight the importance of controlling the ZnO–ZnS composition in biomedical applications.

## 3. Materials and Methods

### 3.1. Synthesis Procedure

#### 3.1.1. Materials

Zinc oxide (ZnO) was prepared by calcination of zinc hydroxycarbonate (2ZnCO_3_·3Zn(OH)_2_·H_2_O, special purity grade) at 1250 °C, followed by cooling in a desiccator over CaCl_2_. Zinc sulfide (ZnS) and antimony(III) sulfide (Sb_2_S_3_) were obtained from JV “New Materials and Technologies” (Odesa, Ukraine). ZnS was synthesized via self-propagating high-temperature synthesis (SHS) from sulfur and metallic zinc powders [49], while Sb_2_S_3_ was produced by fusion of elemental components in a vacuum ampoule. All reagents met “special purity” grade specifications with chromophore impurity levels (Cu, Fe, Cr) below 10^−3^ wt.%.

#### 3.1.2. ZnO-ZnS Synthesis

During SHS, ZnO may partially react with elemental sulfur at >800 K, yielding ZnS and SO_2_. In the subsequent exchange reaction, ZnO and Sb_2_S_3_ can react according to

3ZnO(s) + Sb_2_S_3_(l) ↔ 3ZnS(s) + Sb_2_O_3_(g) ↑ (3)

This reaction is thermodynamically favourable (exothermic, negative entropy change). Continuous evaporation of Sb_2_O_3_ drives the equilibrium toward ZnS formation, enhancing yield.

Mixtures of ZnO and Sb_2_S_3_ were placed in an alumina boat and heat-treated in a high-temperature tubular furnace (RHTC 80-450, Nabertherm, Lilienthal, Germany) under purified argon. Temperature was controlled with ±1 °C accuracy. The chosen regime ensured (i) operation within the liquid phase region of Sb_2_S_3_ (m.p. ~560 °C), (ii) minimal Sb_2_S_3_ vapour pressure (<0.1 mmHg), and (iii) effective volatilization of Sb_2_O_3_ (m.p. ~660 °C) with negligible ZnO/ZnS volatility. Four ZnO–ZnS composites were obtained by adjusting the nominal ZnO:ZnS ratio: ZN_1: 100% ZnO (reference); ZN_2: 99% ZnO + 1% ZnS; ZN_3: 90% ZnO + 10% ZnS; ZN_4: 85% ZnO + 15% ZnS.

### 3.2. Characterization Techniques

The crystal phases were analyzed using an X-ray diffractometer (XRD; D8 Advance, Bruker, Ettlingen, Germany) with Cu K_α_ (α = 0.154056 nm) radiation, operated at 40 kV and 40 mA.

A Helios NanoLab 650 field emission scanning electron microscope (FESEM) (FEI, Hillsboro, OR, USA), operating at 5 kV and 30 kV using an ETD detector in secondary electron imaging mode (SE), was used to obtain images of the morphology of the prepared surfaces. An Energy-Dispersive X-ray Spectroscopy (EDS) method was also applied (EDAX detector, AMETEK, Berwyn, PA, USA). The analysis was carried out over the entire sample area.

The Raman spectra were obtained using an inVia Micro Raman Renishaw spectrometer combined with a Leica DM 2500M microscope (Renishaw, Wotton under Edge, Gloucestershire, UK) equipped with a 488 nm excitation source. The measurements were taken with spot metering mode and map mode. The exposure time was 10 s with triple-scan accumulation and for a laser output power of 2.5 mW. The data were collected in the spectral range of 100–3200 cm^−1^. The measurement was carried out with 20× lens magnification. Baseline correction was performed during data processing.

IR transmission spectra in the range 4000–200 cm^−1^ were recorded on a Frontier PerkinElmer (PerkinElmer Inc., Waltham, MA, USA) spectrometer with Fourier transform. To record the spectrum, the samples were ground with CsI (Ukraine, Institute of single crystals, Kharkiv), high-purity grade, in a ratio of 1:20. The CsI sample was preheated at 180 °C to remove residual moisture.

UV–Vis spectra were recorded using an Agilent Technologies Cary Series UV–Vis-NIR spectrophotometer (Agilent, Santa Clara, CA, USA) in the wavelength range of 200–1200 nm. Finely dispersed MgO served as a comparison sample. The spectral dependences of the Kubelka–Munk function were recorded (Equation (4)):

(4) $$F\left(R\right)=\frac{{\left(1-R\right)}^{2}}{2R}=\frac{k}{s}\;$$

where R is relative reflection; k and s are absorption and scattering coefficients, respectively.

The X-ray photoelectron spectra of samples were measured in a Kratos AXIS Ultra DLD system (Kratos Analytical, Rydalmere, NSW, Australia) equipped with a hemispherical analyzer with a reference intensity of over 50,000 counts per second (cps) and spectral resolution of 0.6 eV, using Al Kalpha1 (1486.6 eV) radiation produced by a monochromatized X-Ray source with spot size of 0.7 mm. The operating power was 144 W (12 kV × 12 mA) and the spectra were recorded with 160 eV pass energy for surveys and 40 eV pass energy for high-resolution measurements, with Hybrid lens mode and slot aperture. Partial charge compensation was achieved using a neutralizer flood gun (filament current of 1.8 A, charge balance of 3 V, filament bias of 2 V). The base pressure in the analysis chamber before measurements was low, 10^−9^ mbar. The above parameters were optimized in order to obtain the C 1s peak of the carbon contamination of the sample at 284.60 ± 0.01 eV. Peaks were resolved with IGOR 9.05 software and assigned by considering reliable literature reports. The spectra were fitted using Voigt peak profiles with the associated inelastic background. The amplitude was corrected with ASF (atomic sensitivity factors) for XPS [50].

The spectra of luminescence and excitation were recorded on a Fluorolog FL3-22 spectrofluorimeter (HORIBA Jobin Yvon Inc., Longjumeau Cedex, France). A 450 W Xenon lamp (model 1907) served as the excitation source. For the visible region of the spectrum, an R928P photomultiplier was used as the radiation detector. The excitation wavelength range was from 240 to 600 nm, and the luminescence wavelength range was from 290 to 850 nm. The photomultiplier registered radiation intensity by counting individual photons of light, maintaining proportionality within a range of 1000 to 2,000,000 counts per second (CPS). Based on this sensitivity range, the input and output slits of the device were adjusted accordingly. The luminescence measurements were conducted in a specialized cuvette for solid powder materials with a depth of 1.5 mm and a surface area of 70 mm^2^. Before recording the luminescence spectrum of the crystalline sample, the sample was ground into a uniform fine powder.

### 3.3. Photocatalytic Testing

Photocatalytic degradation was carried out using a glass photoreactor (Heraeus LRS2, Hanau, Germany) under air conditions. The irradiation was performed with a TQ150 excimer lamp (Heraeus, Hanau, Germany) (150 Watt, of power density 4.7 mW·cm^−2^ measured by a digital lux meter, Peak Tech 5025, which gives light intensity ca. 7.9 × 10^19^ photons per second) immersed in the continuously stirred reaction suspension. In total, 150 mg of catalyst was added to a glass tube reactor containing 250 cm^−1^ of 1 × 10^−5^ mol·dm^−3^ methylene blue (MB) water solution (pH = 5.56 ± 0.02). The suspension was stirred for 30 min in the dark to achieve adsorption/desorption equilibrium. The photocatalytic reaction was continued for up to 60 min. During the reaction, 5 cm^−3^ samples were collected from the reactor at regular time intervals. The concentration of the MB (after removing the catalyst) was determined by spectrophotometric measurements (VWR UV-VIS 3100 PC spectrophotometer, VWR International, Radnor, PA, USA). External standards of five concentrations ranged from 1 × 10^−6^ to 1 × 10^−5^ mol·dm^−3^.

Similar MB photocatalytic degradation tests were conducted in the presence of EDTA (10.0 mM) and t-BuOH (100 mM) as hole scavengers, or KClO_3_ (10.0 mM) as an electron scavenger. All scavenging experiments were performed under an Ar atmosphere to eliminate dissolved oxygen. Prior to catalyst addition, the MB solution was saturated with Ar by continuous bubbling for 30 min in the dark. The pH was adjusted to 6.0 for all scavenging experiments.

### 3.4. Antibacterial Activity Assessment

To assess the antibacterial activity of the four types of nanoparticles, tests were performed on the Staphylococcus aureus strain (ATCC 25923) obtained from the collection of the Microbiology Department of the Medical Faculty of the University of Rzeszów.

The tested nanoparticles (in powder form) included samples ZN_1, ZN_2, ZN_3, and ZN_4. For this research, we used an *S. aureus* strain from overnight culture. The bacteria were cultured in liquid medium (LB broth) at 37 °C with shaking (Benchmark H1001-M Incu-Shaker Mini Compact Shaking Incubator, Benchmark Scientific Inc., Sayreville, NJ, USA). The media (Nutritien broth P-0022, Nutritien agar P-0091) were prepared following the manufacturer’s protocol (BTL Sp. z o.o, Łódź, Poland). Herein, 100 µL of the bacteria suspension was applied on the agar surface, spread by a spreader and left for 15 min to allow absorption into the medium. The next step was applying 20 µL nanoparticles in PBS buffer (2.0 g/mL) in four replicates. PBS was used as the control. The inoculations were performed in a laminar flow cabinet (BioTectum, Bielsko-Biała, Poland). The plates were incubated for 16 h under illumination (wavelength 350–450 nm) at 37 °C and minimum humidity of 85% (Thermo Fisher Scientific Forma Steri Cycle 370 CO_2_ Incubator, Thermo Fisher Scientific Inc., Waltham, MA, USA). The experiment was carried out in three independent replicates.

## 4. Conclusions

ZnO–ZnS nanocomposites with varying ZnS contents were synthesized and comprehensively characterized by XRD, SEM/EDS, Raman, XPS, IR, UV-Vis diffuse reflectance, and photoluminescence spectroscopy. The analyses confirmed the coexistence of crystalline ZnO and ZnS phases, with increasing ZnS content leading to more pronounced surface coverage of ZnO particles and the appearance of characteristic ZnS vibrational and luminescent bands. The optical band-gap values remained nearly constant (3.16–3.18 eV), reflecting the dominance of the ZnO absorption edge in these surface-heterostructured systems.

Photocatalytic experiments using methylene blue revealed that a small ZnS addition (1 wt.%) significantly enhanced photocatalytic activity compared with pure ZnO, whereas higher ZnS contents (10–15 wt.%) reduced activity. This effect is consistent with the band alignment of ZnO–ZnS nanocomposites, which preserves strong reductive electrons and oxidative holes, whereas ZnS-rich compositions lead to suboptimal band alignment and reduced ROS generation.

In the antibacterial assays against *Staphylococcus aureus*, the inhibition zones varied only modestly among the ZnO–ZnS compositions, indicating no pronounced compositional synergy; future work should therefore extend biological testing to additional Gram-positive and Gram-negative strains to broaden the biomedical relevance of these findings. Overall, these results provide key insights for optimizing ZnO–ZnS nanocomposites as multifunctional photocatalysts and antimicrobial agents.

## Figures and Tables

**Table 1:** EDS results for the catalysts.

Sample No.	ZnO Content by Synthesis, %	ZnS Content by Synthesis, %	O Content by EDS, at%	S Content by EDS, at%
ZN_1	100	0	28.4	0.13
ZN_2	99	1	17.09	0.33
ZN_3	90	10	31.38	1.79
ZN_4	85	15	21.53	7.14
ZN_5	0	100	3.91	43.03

**Table 2:** Spectroscopic line frequencies and assignments of Raman signals of catalysts.

Frequencies, cm^−1^						
ZN_1	ZN_2	ZN_3	ZN_4	ZN_5	Line Identification	
100	101	101	101	---	E_2L_	ZnO
205	202	205	216	206	2E_2L_/2LA	ZnO/ZnS
---	280	274	280	273	TO	ZnS
332	333	333	333	---	3E_2H_–E_2L_	ZnO
---	349	351	350	350	LO	ZnS
381	381	381	380	---	A_1_(TO)	ZnO
---	395	393	392	395	TO + LA	ZnS
409	409	410	409	---	E_1_(TO)	ZnO
---	412	415	413	416	TO + TA	ZnS
439	439	439	439	---	E_2H_	ZnO
541	536	539	539	---	E1(TO) + E_2L_	ZnO
584	585	587	582	---	A_1_(LO)/E_1_(LO)	ZnO
---	---	---	---	637	2(TO)	ZnS
656	659	660	655	---	2(E_2H_–E_2L_)	ZnO
---	---	665	670	671	2(LO)	ZnS

**Table 3:** IR transmittance spectra of catalysts.

Content of ZnO, wt.%	Wavenumber, cm^−1^				Intensity of the Main Absorption Band, %
ZN_1	443.3	246.0	229.1	215.3	52.0
ZN_2	442.9	330.3 *	229.1	207.4	65.0
ZN_3	434.9	331.1	279.7 *		70.0
ZN_4	431.8	330.7	275.1	216.9	30.0
ZN_5	415.0	~300.0			25.0

*—inflection.

**Table 4:** Band gaps, conduction band and valence band energy of the ZnO–ZnS system samples.

Sample	Direct Band-Gap Energy, eV	Valence Band Energy, eV	Conduction Band Energy, eV
ZN_1	3.17	2.32	−0.85
ZN_2	3.18	1.35	−1.83
ZN_3	3.18	0.91	−2.27
ZN_4	3.16	1.14	−2.02
ZN_5	3.41	0.77	−2.64

**Table 5:** Maximum band positions of catalysts’ photoluminescence spectra (excitation spectra on the left side, emission spectra on the right side), excitation 380 nm, slits 1–5 nm.

Sample	Maximum Position, nm		Intensity of the Luminescence Maximum, Relative Units × 10^−5^
	Excitation	Emission	
ZN_1	380	564	2.82
ZN_2	380	555	5.33
ZN_3	380	564	6.85
ZN_4	380	593	5.08
ZN_5 *	342	461	4.91

*—SHS product (central region of the sample).

**Table 6:** Values of apparent rate constant of MB degradation at photocatalysts.

Sample	Apparent Rate Constant k_app_ [min^−1^] × 10^−3^	R^2^
ZN_1	61.8	0.97
ZN_2	103.3	0.88
ZN_3	22.2	0.96
ZN_4	22.2	0.96
ZN_5	3.9	1.00
MB photolysis	3.7	1.00

**Table 7:** Parameters of the degradation rate of MB in presence of EDTA, t-BuOH, Ar.

Sample	Apparent Rate Constant k_app_ [min^−1^] × 10^−3^	R^2^
ZN_1	183.8	1.00
ZN_2	185.1	0.98
ZN_3	132.5	1.00
ZN_4	130.7	1.00
ZN_5	155.2	1.00

**Table 8:** Parameters of the degradation rate of the MB in the presence of KClO_3_, Ar.

Sample	Apparent Rate Constant k_app_ [min^−1^] × 10^−3^	R^2^
ZN_1	34.3	0.99
ZN_2	31.7	1.00
ZN_3	19.1	1.00
ZN_4	18.9	0.99
ZN_5	6.9	0.99

## Data Availability

The data supporting this article have been included as part of the Supplementary Information.

## Supplementary Materials

The following supporting information can be downloaded at https://www.mdpi.com/article/10.3390/molecules31061010/s1: Figure S1: EDS spectra of samples of the ZnO–ZnS system: a—ZN_1; b—ZN_2; c—ZN_3; d—ZN_4; e—ZN_5. Figure S2: Absorption spectra of catalysts. Figure S3: Raman mapping of ZN_1 (a), ZN_2 (b), ZN_3 (c), ZN_4 (d), ZN_5 (e) (λ = 488 nm). Figure S4: Survey spectra of the samples (ZN_1—black; ZN_2—red; ZN_3—blue; ZN_4—green; ZN_5—yellow). Figure S5: Zn LMM high-resolution X-ray photoelectron spectroscopy (XPS) measurements on samples. Figure S6: High-resolution X-ray photoelectron spectroscopy (XPS) measurements on samples ZN_1-ZN5 (a, b, c, d, e respectively). The experimental data are shown as red dots/lines, while the fit is represented by the black line. Zn 2p was fitted with two components (Zn(OH)_2_-like and lattice Zn^2+^) in ZN_1–ZN_4 and one component in ZN_5 and S 2p was fitted with two components in ZN_5 and one component in ZN_1-ZN4. Figure S7: XPS valence band spectra of ZN_1 (a), ZN_2 (b), ZN_3 (c), ZN_4 (d), ZN_5 (e). Figure S8: Photoluminescence spectra of catalysts excited at different wavelengths: ZN_2 (a); ZN_3 (b), ZN_4 (c), ZN_5 (d). Table S1: Thermodynamic characteristics of the double ion exchange reaction (1) of ZnO sulfidation. Table S2: The C 1s, O 1s, Zn 2p, S 2p intensities. Table S3: Binding energies of Zn 2p_3/2_; ZnLMM Auger line binding and kinetic energies; Auger parameter and assignments for the 5 samples. Component 2 corresponds to lattice Zn^2+^ in ZnO or at the ZnO–ZnS interface, whereas component 1 corresponds to surface-hydroxylated ZnO or Zn(OH)_2_-like species.

## Abbreviations

The following abbreviations are used in this manuscript:

SHS	Self-propagating high-temperature synthesis
XRD	X-ray Diffraction
FESEM	Field emission scanning electron microscope
EDS	Energy-Dispersive X-ray Spectroscopy
MB	Methylene blue
