# Supplementary material for: Composition-Controlled Photocatalytic and Antibacterial Performance of ZnO-ZnS Nanocomposite Catalysts Synthesized by Solid-State Ion Exchange
Source: Molecules. 2026 Mar 17;31(6):1010. doi: 10.3390/molecules31061010 (PMC13029210; doi:10.3390/molecules31061010)
Supplement: Supplementary file 1 [file molecules-31-01010-s001.zip › molecules-4149446-supplementary.pdf]

Supplementary Materials

Composition Controlled Photocatalytic and Antibacterial Performance of ZnO-ZnS Nanocomposite Catalysts Synthesized by Solid-State Ion Exchange

Joanna Wojtas[1], Viktor Zinchenko[2], Renata Wojnarowska-Nowak[3], Anna Żaczek[1], Igor Magunov[2], Pavlo Doga[2], Anton Babenko[4], Sergii Pavlov[5], Yaroslav Bobitski[3], Joanna Kisała[3,*]

1 Faculty of Medicine, Collegium Medicum, University of Rzeszow, Kopisto 2a Ave., 35-315 Rzeszow, Poland; jowojtas@ur.edu.pl (J.W.); azaczek@ur.edu.pl (A.Z.)
2 A.V. Bogatsky Physico-Chemical Institute of National Academy of Sciences of Ukraine, Odesa, Ukraine; vfzinchenko@ukr.net (V.F.Z.); igmagua@ukr.net (I.R.M.); dogapavel@gmail.com(P.G.D.)
3 Faculty of Exact and Technical Sciences, University of Rzeszow, Pigonia 1 Str., 35-310 Rzeszow, Poland; rwojnarowska@ur.edu.pl (R.W.N.); ybobytskyy@ur.edu.pl (Y.B.); jkisala@ur.edu.pl (J.K.)
4 Faculty of Chemistry and Pharmacy, Odesa I.I. Mechnikov National University, Odesa, Ukraine; anton.octane.sr@gmail.com (A.V.B.)
5 Dept. of Biomedical Engineering and Optic-Electronic Systems Vinnytsia National Technical University, Ukraine; psv@vntu.edu.ua (S.P.)
* Correspondence: jkisala@ur.edu.pl (J.K.)

A preliminary prediction of possible double exchange reactions was made using the concept of acidity-baseness. It is believed that they occur according to the scheme:

$$3ZnO(s) + Sb_2S_3(l) \leftrightarrow 3ZnS(s) + Sb_2O_3(g)\uparrow. \quad (1)$$

It has been established that Zn^{2+} and Sb^{3+} ions belong to the group of acids of intermediate type, as well as O^{2-} and S^{2-} ions belong to hard and soft bases, respectively, in terms of softness and hardness according to Pearson. Therefore, the exchange reaction has a little effect on the nature of the «soft-hard» combination.

The given prediction is confirmed by the data of thermodynamic calculations of the reaction. As can be seen from Table S1, the indicated reaction (1) is exothermic and proceeds with a negative change in entropy. It should be noted that the evaporation of Sb_2O_3 contributes to the shift of the equilibrium to the right side, and therefore to the increase of the efficiency of the process.

Table S1. Thermodynamic characteristics of the double ion exchange reaction (1) of ZnO sulfidation.

Compound	ΔH°_T, kJ/mole		S°_T, J/mole·K		ΔH°_r, kJ/mole	ΔS°_r, J/mole·K	ΔG°_r, kJ/mole
	ΔH°_{298}	ΔH°_{873}	S°_{298}	S°_{873}			
ZnO	–350.5	–320.8	43.6	99.35			
Sb_2S_3	–205.0	–80.8	182.0	381.3			
ZnS (sphalerite)	–205.0	–172.7	57.7	112.7			
Sb_2O_3 (rhombic)	–708.6	–650.3	123.0	232.0			
The reaction (1), T = 873 K					–125.2	–109.3	–29.8

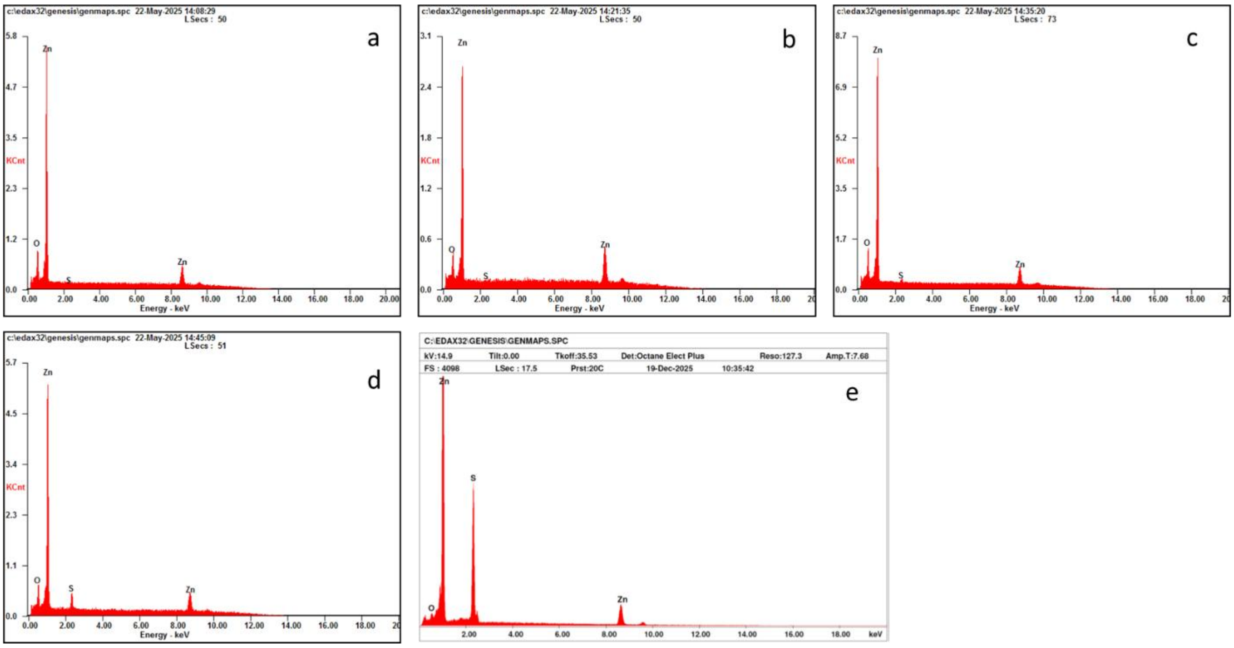

Fig. S1. EDS spectra of samples of the ZnO–ZnS system: a – ZN_1;b – ZN_2, c-ZN_3, d-ZN_4, e-ZN_5.

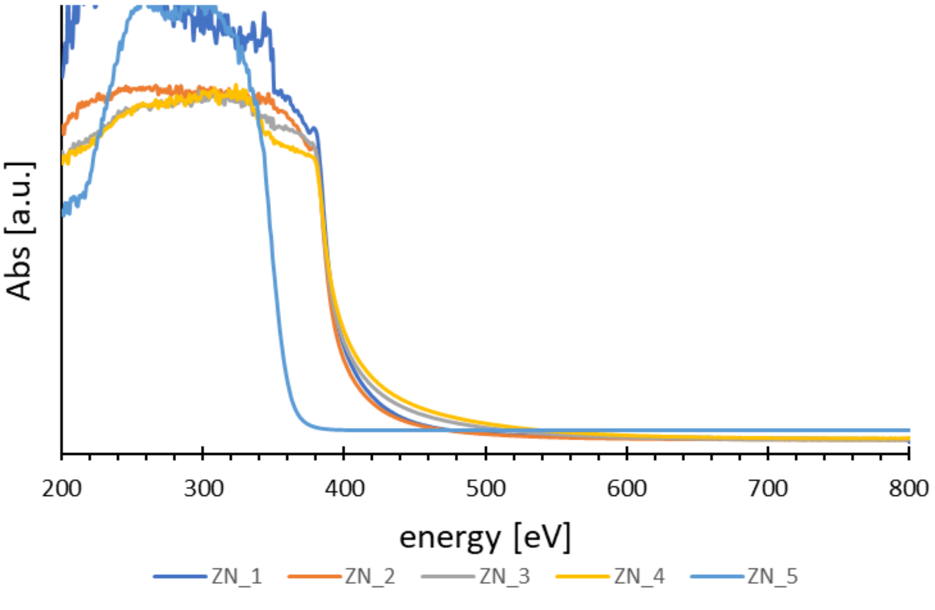

Fig. S2. Absorption spectra of catalysts.

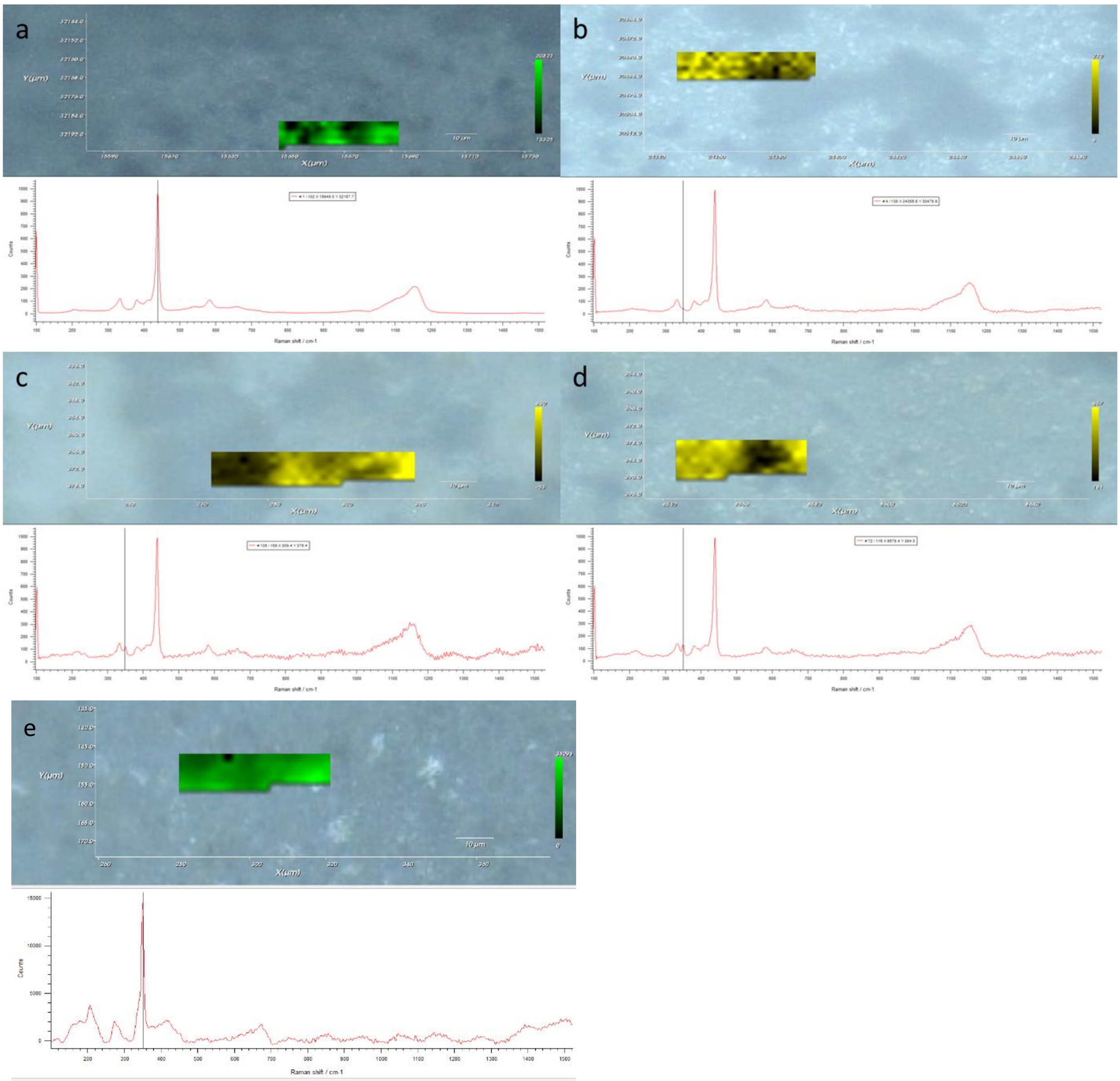

Fig. S3. Raman mapping of ZN_1 (a), ZN_2 (b), ZN_3 (c), ZN_4 (d), ZN_5 (e) (λ = 488 nm).

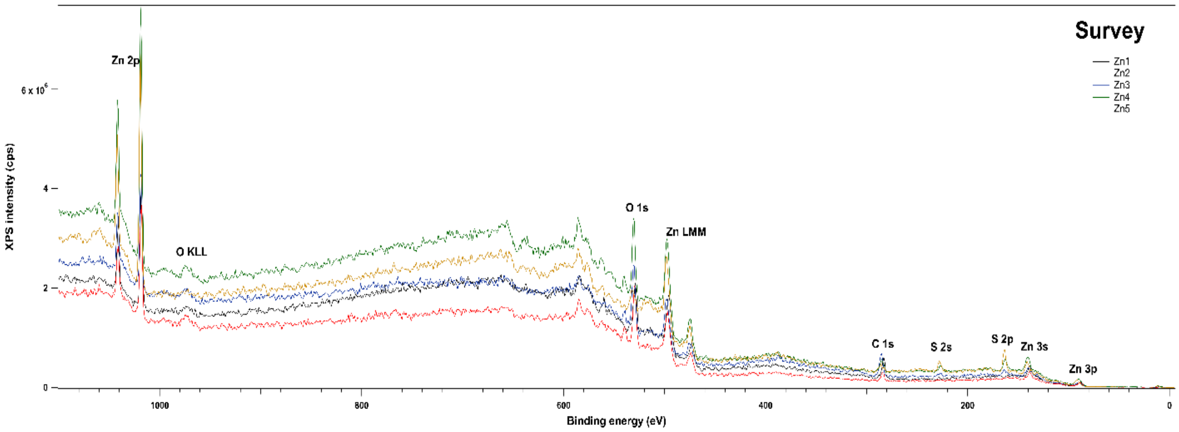

Fig. S4. Survey spectra of the samples (ZN_1-black, ZN_2-red, ZN_3-blue, ZN_4-green, ZN_5-yellow).

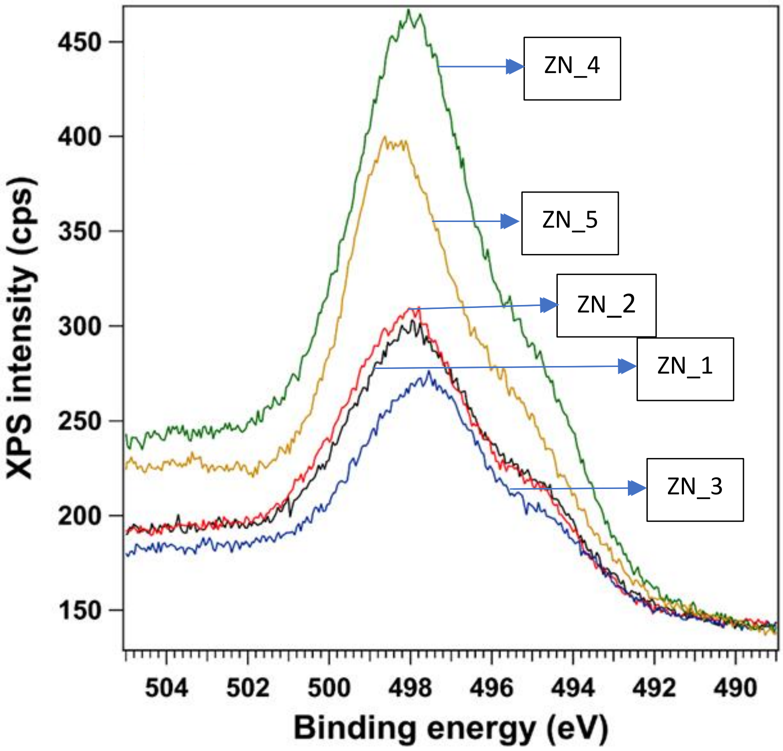

Fig. S5. Zn LMM high-resolution X-ray photoelectron spectroscopy (XPS) measurements on samples.

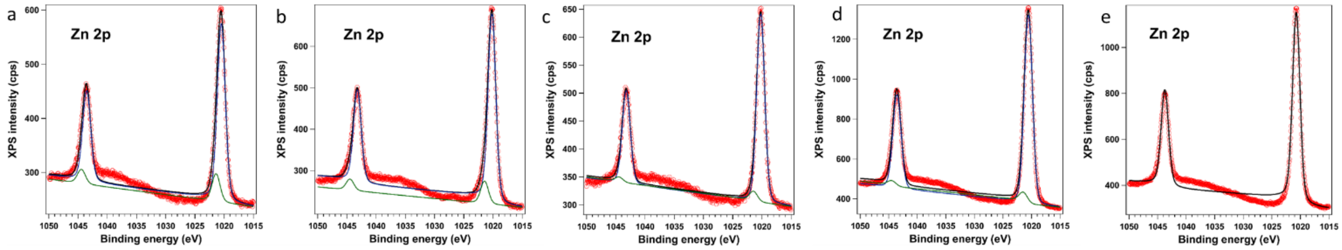

Fig. S6. High-resolution X-ray photoelectron spectroscopy (XPS) measurements on samples ZN_1-ZN5 (a, b, c, d, e – respectively). The experimental data are shown as red dots/lines, while the fit is represented by the black line. Zn 2p was fitted with two components ($Zn(OH)_2$-like and lattice Zn^{2+}) in ZN_1–ZN_4 and one component in ZN_5 and S 2p was fitted with two components in ZN_5 and one component in ZN_1-ZN4.

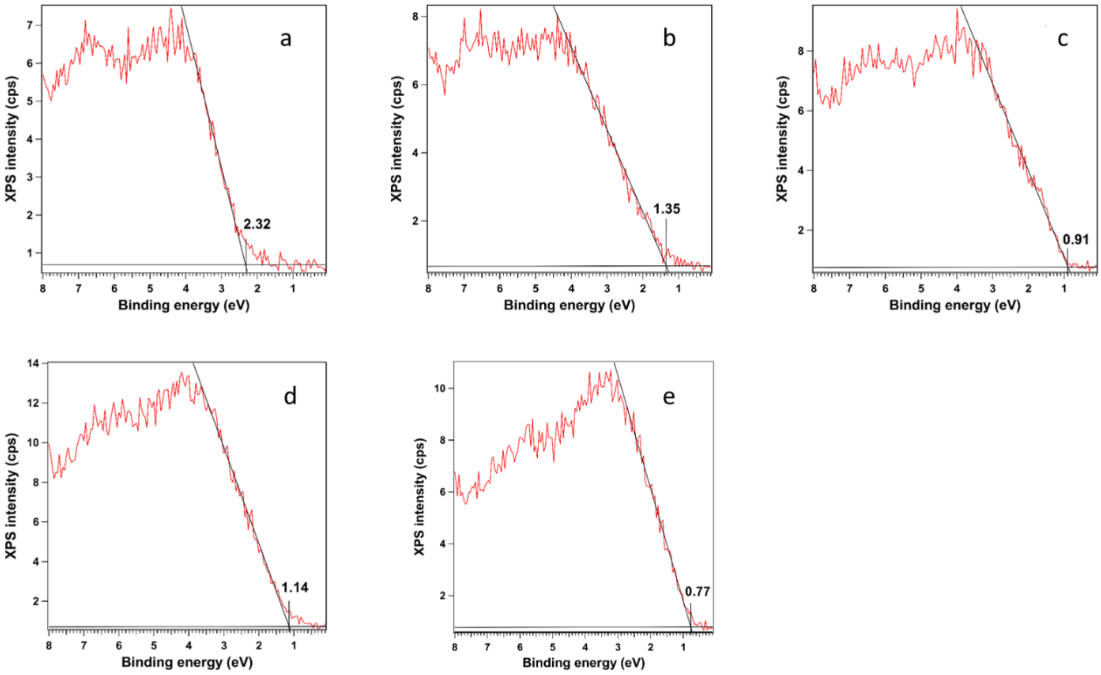

Fig. S7. XPS valence band spectra ZN_1 (a), ZN_2 (b), ZN_3 (c), ZN_4 (d), ZN_5 (e).

In the PL spectra presented in this article, we used light with a wavelength of 380 nm to excite luminescence, as this wavelength corresponds to the maximum of the excitation spectrum and is also where the maximum luminescence intensity is observed (at a wavelength of 560-585 nm). When using a non-optimal excitation wavelength (300, 320, 350, 365, 390 nm), the maximum of the luminescence band of the ZnO–ZnS system (at a wavelength of 560-585 nm) does not shift, but becomes significantly lower in intensity. However, a luminescence band (peaking at 386 nm) is observed in the 365-405 nm range, but it is very weak in intensity. Excitation spectra for the 386 nm luminescence band were also recorded. Their shape resembles the excitation spectrum for the luminescence band at λ = 560 nm and does not reveal any new excitation bands in the 250-370 nm range. In the luminescence spectrum of ZnS, when using shorter-wavelength excitation radiation (λ = 295, 330, 342 nm) than the optimal wavelength (λ = 357 nm), no additional luminescence bands are observed in the 350-400 nm range, but the position of the luminescence band maximum shifts.

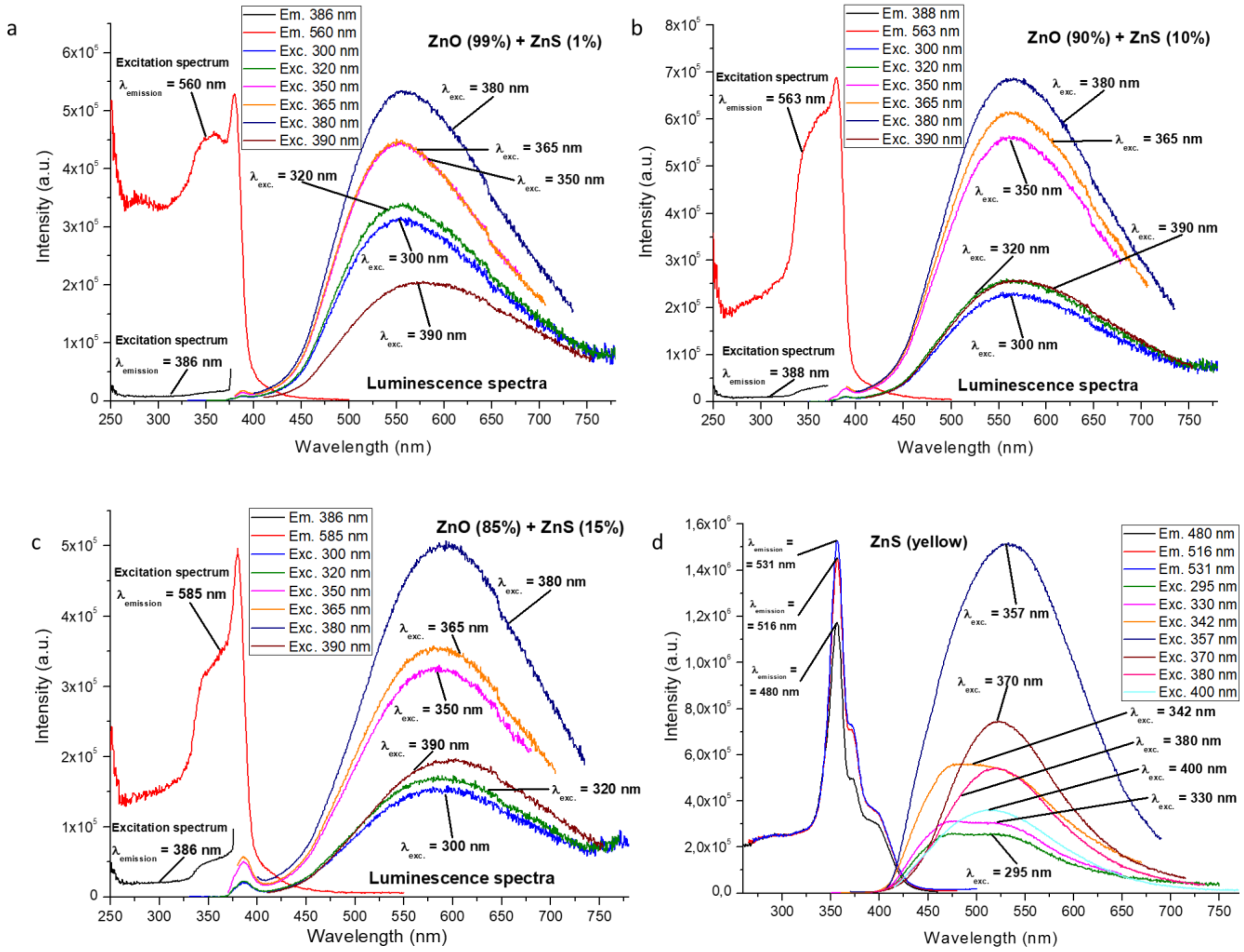

Fig. S8. Photoluminescence spectra of catalysts excited at different wavelengths ZN_2 (a); ZN_3 (b), ZN_4 (c), ZN_5 (d).

Table S2. The C 1s, O1s, Zn 2p, S 2p intensities.

	ZN_1	ZN_2	ZN_3	ZN_4	ZN_5
	Intensity(cps)	Intensity(cps)	Intensity(cps)	Intensity(cps)	Intensity(cps)
C 1s	2728.2	1109.6	3614.1	2136.8	1556.4
O 1s	746.3	952.8	1104.4	1289.3	513.2
Zn 2p	229.1	287.9	201.5	584.8	488.0
S 2p	17.6	42.6	201.8	625.2	1371.2

Table S3. Binding energies of Zn $2p_{3/2,}$ ZnLMM Auger line in binding and kinetic energiers, Auger parameter and assignements for the 5 samples. Component 2 corresponds to lattice Zn^{2+} in ZnO or at the ZnO–ZnS interface, whereas component 1 corresponds to surface-hydroxylated ZnO or $Zn(OH)_2$-like species.

	Zn $2p_3/_2$ binding energy (component 1)	Zn $2p_3/_2$ binding energy (component 2)	BE_{ZnLMM}	KE_{ZnLMM}	Auger parameter for component 1	Assign component 1	Auger parameter for component 2	Assign component 2
ZN_1	1020.49	1021.39	497.96	988.64	2009.13	surface $Zn(OH)_2$/ hydroxylated ZnO	2010.03	lattice Zn^{2+} in ZnO or ZnO–ZnS interface
ZN_2	1020.19	1021.46	497.99	988.61	2008.8	surface $Zn(OH)_2$/ hydroxylated ZnO	2010.07	lattice Zn^{2+} in ZnO or ZnO–ZnS interface
ZN_3	1020.21	1021.43	497.64	988.96	2009.17	surface $Zn(OH)_2$/ hydroxylated ZnO	2010.39	lattice Zn^{2+} in ZnO or ZnO–ZnS interface
ZN_4	1020.55	1021.41	498.00	988.6	2009.15	surface $Zn(OH)_2$/ hydroxylated ZnO	2010.01	lattice Zn^{2+} in ZnO or ZnO–ZnS interface
ZN_5	1020.72	-	498.37	988.23	2008.95	surface $Zn(OH)_2$/ hydroxylated ZnO	-	-
